# Transmural collaborative care model for the review of antipsychotics: a feasibility study of a complex intervention

**DOI:** 10.1038/s41598-024-62349-9

**Published:** 2024-05-29

**Authors:** Kirsti M. Jakobs, Karlijn J. van den Brule-Barnhoorn, Jan van Lieshout, Joost G. E. Janzing, Wiepke Cahn, Maria van den Muijsenbergh, Marion C. J. Biermans, Erik W. M. A. Bischoff

**Affiliations:** 1 Primary and Community Care Department Nijmegen, Radboud University Medical Center, Nijmegen, The Netherlands; 2 Zorggroep Onze Huisartsen, Arnhem, the Netherlands; 3 IQ Health Science Department, Radboud University Medical Center, Nijmegen, The Netherlands; 4 Psychiatry Department, Radboud University Medical Center, Nijmegen, The Netherlands; 5 Psychiatry Department, University Medical Center Utrecht, Utrecht, The Netherlands; 6 Pharos, Dutch Centre of Expertise On Health Disparities, Utrecht, The Netherlands

**Keywords:** Health care, Risk factors

## Abstract

General practitioners (GPs) are often unaware of antipsychotic (AP)-induced cardiovascular risk (CVR) and therefore patients using atypical APs are not systematically monitored. We evaluated the feasibility of a complex intervention designed to review the use of APs and advise on CVR-lowering strategies in a transmural collaboration. A mixed methods prospective cohort study in three general practices in the Netherlands was conducted in 2021. The intervention comprised three steps: a digital information meeting, a multidisciplinary meeting, and a shared decision-making visit to the GP. We assessed patient recruitment and retention rates, advice given and adopted, and CVR with QRISK3 score and mental state with MHI-5 at baseline and three months post-intervention. GPs invited 57 of 146 eligible patients (39%), of whom 28 (19%) participated. The intervention was completed by 23 (82%) and follow-up by 18 participants (64%). At the multidisciplinary meeting, 22 (78%) patients were advised to change AP use. Other advice concerned medication (other than APs), lifestyle, monitoring, and psychotherapy. At 3-months post-intervention, 41% (28/68) of this advice was adopted. Our findings suggest that this complex intervention is feasible for evaluating health improvement in patients using AP in a trial.

## Introduction

Care for patients using antipsychotics (APs) is complex, and general practitioners (GPs) have become increasingly involved in this care. They participate in a growing trend of initiating APs off-label, e.g. for anxiety, personality disorders, or sleeping problems^1–4^. In 55% of the cases in the Netherlands, APs are prescribed by GPs^5^.

Mainly atypical APs have been shown to increase cardiovascular risk (CVR)^6,7^. Patients using APs should be monitored at least annually to find and treat adverse effects according to international guidelines^8–11^. In many countries, such as the UK and the Netherlands, in primary care, chronic disease management programs have been developed for CVR management (CVRM)^12,13^. In these programs, trained nurses help patients to reduce CVR with lifestyle interventions and medication.

However, patients on APs are rarely included in CVRM programs^14,15^. In our earlier study, examining the facilitators and barriers for CVRM for patients with severe mental illness (SMI) and/or APs, GPs mentioned several barriers, including a lack of awareness of the elevated risk, reluctance to invite these patients to their program as this could be complicated and time-consuming, and low expectations on the capability of these patients to develop a healthy lifestyle^14^. GPs stated that they feel responsible for their patient’s health, but that changes to the APs should be the responsibility of the psychiatrist^14^.

Papers about the efficacy of interventions to lower the CVR of patients with SMI and/or APs in primary care are scarce. Only one comprehensive trial, Primrose, studied this among patients with an SMI, high levels of cholesterol, and one other risk factor^16^ but found no difference on total cholesterol level at 12 months follow-up.

We think that a transmural intervention in which the GP is supported by a psychiatrist about considering specific AP side effects and interactions can raise the efficacy. For instance, dose reduction and switching to an AP drug with a better metabolic profile are promising strategies to lower CVR. The intervention must help to overcome the barriers mentioned by GPs and address relevant patient factors, which may hinder the required personalization of CVRM.

**Figure 1:**
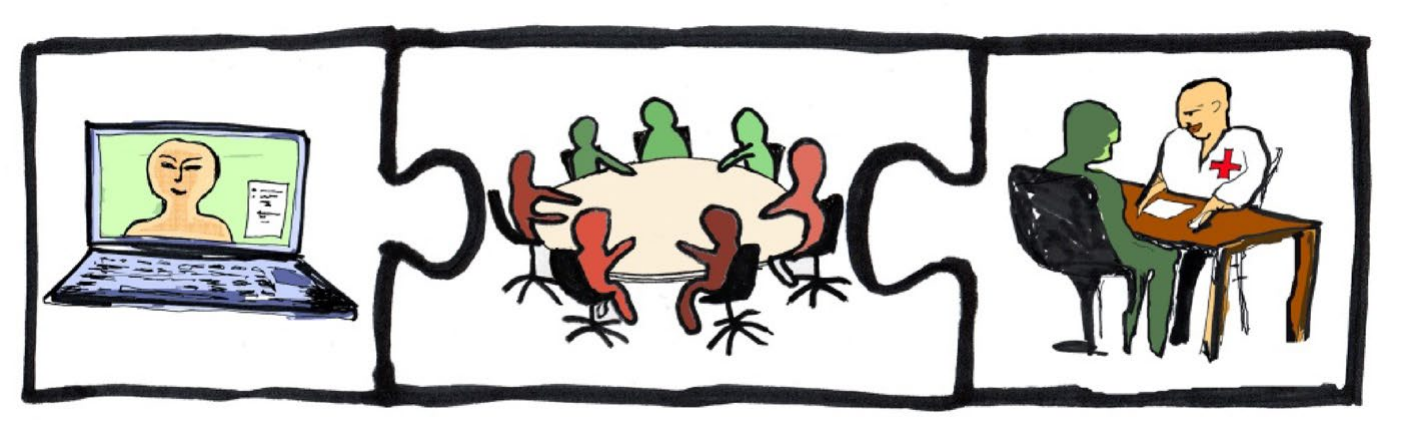
The TACTIC intervention consists of a webinar, a multidisciplinary meeting, and a shared decision-making visit.

To tailor care to patients’ specific needs, a complex intervention was developed by a regional transmural task force consisting of relevant stakeholders, e.g. GPs, psychiatrists, nurses, people with lived experience, and pharmacists (see project description and figure S1 in the supplements)^17^. This intervention is called ‘**T**ransmural collaborative care model for CVRM and medication review for patients using **A**ntipsy**C**ho**TIC**s (TACTIC)’ (Fig. 1). After completing TACTIC, both patients and professionals are better prepared to follow the regular CVRM program in the general practice.

Authors of a recently published review reported a paucity of papers on conducting AP medication reviews in primary care^18^. TACTIC meets the recommendations made by the authors for such an intervention: to foster conversations between GPs and patients, to increase knowledge regarding AP treatment, and enable appropriate and safe prescribing^18^.

TACTIC is a complex intervention, as defined by the British Medical Research Council^19^, and, therefore, conducting a mixed methods feasibility study before conducting a trial is recommended. The results of a comprehensive qualitative study will be reported separately. If proven feasible, the potential effects of TACTIC on CVR and mental health in patients using APs will be studied in a future stepped-wedge cluster randomized controlled trial, which has been planned for 2023 and 2024 (clinicaltrials.gov NCT05647980). The main objective of this quantitative feasibility study was to evaluate the delivery of TACTIC, including recruitment and retention of subjects. Secondary objectives were to outline the baseline characteristics of this patient group; to explore the numbers and types of advice given regarding the use of APs and CVR during the multidisciplinary meetings; and a preliminary examination of the effectiveness.

## Methods

### Ethics

Ethical approval for this study was waived by the local Medical Research Ethics Committee Arnhem/Nijmegen (file number 2020-7240). This study was conducted according to Dutch legislation on privacy and the Declaration of Helsinki. All patients were properly informed and gave written informed consent.

### Study design

In 2021, we conducted a prospective cohort feasibility study in which we implemented the TACTIC intervention in three Dutch general practices and followed participants for 3 months after they received the intervention. Reporting is in line with the CONSORT extension for randomized pilot and feasibility trials^20,21^.

### Setting

Three practices were approached and agreed to participate. These practices are members of the primary care cooperative ‘Onze Huisartsen’, located in the Eastern part of the Netherlands, which united 105 general practices with 385,408 registered patients at the time of the study. Of these patients, 4,045 (1.05%) were ≥ 25 years of age and used APs.

### Participants

The criteria for patient inclusion and exclusion are shown in Table 1. In our future trial, we intend to assess our primary outcome CVR using the QRISK3 score (see the explanation of the QRISK3 algorithm in the ‘Data analysis’ section). The QRISK3 algorithm is only valid for people who do not already have a diagnosis of cardiovascular disease (CVD; coronary heart disease or stroke/transient ischemic attack). Therefore, a history of cardiovascular diseases is one of the exclusion criteria of our study.

**Table 1:** In- and exclusion criteria.

Inclusion criteria
Chronic use of AAPs, defined as ≥3 prescriptions or ≥2 repeat prescriptions or a label for chronic use. The ATC codes* are similar to those in the QRISK3** algorithm as far as they are registered in the Netherlands: N05AX12, N05AD06, N05AH02, N05AE05, N05AH03, N05AX13, N05AH04, N05AX08, N05AE03, N05AX15, N05AX16 Under care of the GP for mental disorder. First, this was defined as “not under care of a psychiatrist” based on the lack of correspondence in the patient’s electronic medical record in the past 12 months. However, it appeared that correspondence was often missing even though the patient was still seeing a psychiatrist. Therefore, we changed the definition to: “the GP authorized the renewal of AAP prescriptions” and is therefore responsible for monitoring the pros and cons.
Exclusion criteria
Age <25 or >84 years. The QRISK3** algorithm is not validated for these age groups A history of CVD, signalled by the ICPC codes*** K74, K75, K76, K77, K90.00, K90.03, K92.01, and K99.01. QRISK3** can only be used for patients without CVD A diagnosis of delirium or dementia (ICPC codes*** P15.02, P70 or P71). The execution of the TACTIC intervention is unsuitable for patients on AAP for these diagnoses.

*AAP *atypical antipsychotic, *ATC *anatomical therapeutic chemical, *CVD *cardiovascular disease, *CVRM* cardiovascular risk management, *GP* general practitioner, *ICPC* International Classification of Primary Care.

*The prescriptions from the ATC codes^22^.

**QRISK3 is a tool to calculate a person's risk of developing a CVD over the next 10 years^12^.

***The diagnoses from the ICPC codes^23^.

Each GP generated a list of eligible patients based on the electronic medical records (EMRs)^24^. The list included all patients meeting the criteria as described in Table 1. To exclude patients under psychiatric care, GPs had to check for any correspondence. However, the GPs informed us that, during the process of inviting patients, many times correspondence was lacking when a psychiatrist was involved. Therefore, we changed the definition of our inclusion criterion ‘Under care of the GP for mental disorder’ from ‘not under care of a psychiatrist’ to ‘the prescriber of the AP must be the GP’. After all, the prescriber is responsible for monitoring adverse effects.

We expected to include 84 eligible patients in three practices, based on the average number of AP users in Dutch general practices^5^ and the number of registered patients in the participating practices. This amount is enough to evaluate the delivery of TACTIC and will show how many practices we need to include to reach the preferred sample size in our future trial.

GPs invited the selected patients by telephone in the period March to May 2021. In case patients were interested, further information about the study was sent to them by mail. Study information was tailored to readers with a low literacy level. Each patient was then called by members of the research team (KMJ or KJvdBB) to answer possible questions and check the study criteria. All patients who were willing to participate signed informed consent and were invited to their general practice for a baseline assessment before the TACTIC intervention started. Details of the baseline assessment will be described later on.

### TACTIC intervention

TACTIC comprised three unique and consecutive steps (also see Fig. 1):

- Step 1. A 90-min digital group meeting to inform patients and their close ones about the multidisciplinary meeting in Step 2. We used an online tool called WebinarGeek, in which patients could join anonymously, chat live, and replay the recordings^25^. During the webinar, the individuals with whom the patient would interact during the multidisciplinary meeting introduced themselves and clarified their roles. This was particularly essential for the patient coach with lived experience and the nurse since patients were not aware of how they could benefit from their assistance. After the webinar, and as an extra preparation for the next step, each patient’s pharmacist provided information on medication use and interactions. In the Netherlands, patients are free to choose their preferred pharmacy. However, they don't often switch pharmacies as only 1.9% of patients receive medication from a different pharmacy than the one they used in the previous year^26^. All relevant information was shared with the psychiatrist using digital consultation^24^; this included diagnoses, medication, blood pressure, body mass index (BMI), laboratory results on CVR, pharmacist medication review, results of the side effects questionnaire, and the most recent psychiatrist’s letter (if available).
- Step 2. A 15-min multidisciplinary meeting per patient. The time allotted for individual meetings was considered to be enough and an efficient use of all caregivers' time. At the meeting, the patient, a caregiver (optional), the GP, a psychiatrist, a nurse specialized in CVRM or mental health, and a patient coach with lived experience evaluated the patient’s medication and CVR. The role of the coach was to underline the patient’s perspective and to introduce sources of support within the community to improve their well-being^27^. The multidisciplinary meeting resulted in individualized advice on AP use (continuation, deprescribing, or switching) and reducing CVR by lifestyle strategies and possibly medication.
- Step 3. A visit to the GP in which the advice of Step 2 was used to draw up an individualized treatment plan by shared decision-making.

Three months after receiving the TACTIC intervention, all participants were invited for a follow-up visit with the nurse for measurements and to evaluate the plan.

### Outcome measurements

For our main objective, i.e. to evaluate the delivery of TACTIC, including recruitment and retention of subjects, we collected at three months follow-up the following information. The GPs manually added whether they invited each patient, and reasons for non-invitations or non-participations, to an anonymized list of eligible patients that was received by the research team via secured email. After obtaining informed consent, patients visited their GP for baseline measurements (T0). Their participation in TACTIC was documented in the EMR, including dates, advice, and plans.

For our secondary objective 'to outline the baseline characteristics of this patient group' we collected from the EMR of each practice the following CVR measures: BMI, systolic blood pressure (including variability), lipid measurements, estimated glomerular filtration rate, albumin-to-creatinine ratio, and smoking status. Moreover, all actual diagnoses relevant for inclusion and exclusion and estimation of CVR, all prescriptions of the past 5 years, relevant referrals, financial records indicative of socioeconomic status (in the Netherlands, per capita rates are higher in deprived areas), and recorded advice and treatment plans concerning TACTIC were collected.

#### Data from questionnaires

Participants completed the following digital questionnaires at baseline (a total of 37 questions):

- Questions about smoking habits, achieved education level (low, middle, or high)^28^, ethnicity, and family history of CVD.
- The Somatic Mini Scale (SMS), based on the Liverpool University Neuroleptic Side Effect Rating Scale, in which patients score 18 adverse effects of their medication on a five-point Likert scale. The score ranges from 0 to 72. This questionnaire was developed by Mental Health Services Central, a community mental health service provider in the Netherlands, and is in the process of validation. According to Mental Health Services Central, a score of 30 or higher is hazardous and should be reported to the prescriber^29^.
- The Mental Health Inventory (MHI-5), a subscale of the 36-item Short-Form Health Survey, to measure mental health-related quality of life^30^. The score is between 0 and 100, and patients with a score ≥ 60 are considered mentally healthy.
- The EuroQol 5 dimensions 5 levels (EQ-5D-5L) questionnaire, to measure the generic quality of life^31^. These five questions were included to enable us to compute quality-adjusted life years in the future trial. The scores range from less than 0 (where 0 is the value of a health state equivalent to death; negative values represent values as worse than death) to 1 (the value of full health).

For our secondary objective 'to conduct a preliminary analysis of the effectiveness', at the 3-month follow-up visit with the practice nurse, we collected CVR measures (T1) from the EMR. The questionnaires were repeated and supplemented with the Dutch-validated 8-item Client Satisfaction Scale (CSQ-8)^32^. The latter is recommended for use in psychiatric patients to measure patients’ satisfaction with care^32^. The sum of eight sub-scores about different aspects of therapy (TACTIC) can vary between 8 and 32, with higher scores indicating greater satisfaction.

### Data analysis

The data were examined using descriptive statistics. Means and standard deviations (SDs) were calculated for continuous data, and frequencies and percentages were calculated for categorical data. For the analysis of changes in QRISK3 score, we used the Wilcoxon signed-rank test in SPSS (version 25).

#### QRISK3 algorithm

To assess CVR, the Dutch guideline advices to use SCORE^13^. We opted for QRISK3 over SCORE because SCORE fails to take into account the extra risk that comes with an SMI or the use of APs^33^, and it is not validated to evaluate the risk of patients who suffer from diabetes. For patients with diabetes, the predicament involving the use of antipsychotic is even more pressing than for those who do not have diabetes. Therefore, we used QRISK3^34^, which does include diabetes and the aforementioned additional risks for this patient group. QRISK3 is designed as a screening tool. We had to make adjustments to the QRISK3 score algorithm to enable us to measure change. These adjustments are found in Table S1 in the supplements. The Townsend deprivation score (TDS) is one of the variables of the QRISK3 score. In the Netherlands, a different deprivation index is used^35,36^. In the QRISK3 score algorithm, the TDS was set to zero because we did not have this information. Additionally, we applied a revised TDS score and reported this as QRISK3_TDS. To avoid overestimation of the risk, we used the TDS value at p20 (below which are the 20% most deprived of the British population) for the 10% most deprived patients in the Dutch population, who are identified in the financial EMRs, which are based on postal codes stratified by measuring three variables: wealth, level of education, and unemployment^37^.

#### Additional analyses of QRISK3 score

In absolute risk assessments like QRISK3 the influence of unmodifiable CVR factor like age is high. To gather more insight about what can be gained in health improvement for this often overlooked patient group, we wanted to explore different outcome measures to show health effects for the individual rather than the mean changes. Therefore, we calculated the room for improvement for each individual (qrisk_max_achievable_reduction), which is the difference from a QRISK3 score of a person with all modifiable risk factors optimized. Furthermore, we calculated the proportional risk reduction by using this formula: (qrisk3_score_T0—qrisk3_score_T1) / qrisk_max_achievable_reduction) * 100 (Table 2).

The proportional risk reduction expresses that a patient with 10% risk, who could improve to 5% has a maximum of 5% risk reduction. An improvement of 1% would be a proportional risk reduction of (1:5 *100 =) 20%.

**Table 2:** Participant characteristics at baseline (n = 28).

Demographic	
Age in years mean (SD)	49 (11.1)
Female n (%)	13 (46.4)
Country of birth	
The Netherlands n (%)	24 (85.7)
Morocco n (%)	2 (7.1)
Other n (%)	2 (7.1)
Education*	
Low n (%)	7 (22.2)
Middle n (%)	11 (40.7)
High n (%)	10 (37.0)
Low socioeconomic status n (%)	10 (35.7)

Mental health	
Primary psychiatric disorders	
Depressive disorder n (%)	6 (21.4)
Personality disorder n (%)	5 (17.9)
PTSD n (%)	4 (14.3)
Autistic spectrum disorder n (%)	3 (10.7)
Anxiety disorder n (%)	3 (10.7)
Bipolar disorder n (%)	2 (7.1)
Psychosis n (%)	2 (7.1)
Anorexia nervosa n (%)	1 (3.6)
ADHD n (%)	1 (3.6)
Insomnia n (%)	1 (3.6)
AP agent	
Quetiapine n (%)	16 (57.1)
Risperidone n (%)	7 (25.0)
Aripiprazole n (%)	3 (10.7)
Olanzapine n (%)	2 (7.1)
Adverse effects for APs	
Not at all	0
Very little	0
A little n (%)	3 (10.7)
Much n (%)	16 (57.2)
Very much n (%)	9 (32.1)
MHI-5 score mean (SD)	56.79 (18.01)

Quality of life:	
EQ-5D score mean (SD)	0.31 (0.28)
CVR:	
QRISK3 score mean (SD)	11.17 (14.51)
QRISK3 score with revised TDS mean (SD)	11.89 (14.85)
Smoking**	
Never n (%)	8 (28.6)
Past n (%)	10 (35.7)
Light n (%)	4 (14.3)
Medium n (%)	5 (17.9)
Heavy n (%)	1 (3.6)
Atrial fibrillation n (%)	1 (3.6)
Migraine n (%)	3 (10.7)
Chronic kidney disease, stages 3–5 n (%)	4 (14.3)
Family history of CVD n (%)	13 (46.4)
Diabetes mellitus type 2 n (%)	2 (7.1)
Chronic corticosteroids n (%)	2 (7.1)
Statins n (%)	1 (3.6)
Antihypertensive medication n (%)	4 (14.3)

*ADHD* attention deficit hyperactivity disorder, *AP* antipsychotic, *CVD* cardiovascular disease, *CVR* cardiovascular risk, *EQ-5D* generic quality of life, *MHI* Mental Health Inventory, *PTSD* post-traumatic stress disorder, *QRISK* person's risk of developing a heart attack or stroke over the next 10 years, *SD* standard deviation, *TDS* Townsend deprivation score.

*Definition of grouping according to Central Bureau voor de Statistiek (Statistics Netherlands)^28^.

**Light smoker < 10, moderate 10–19, heavy > 19 cigarettes a day.

## Results

### Recruitment and retention of subjects

**Figure 2:**
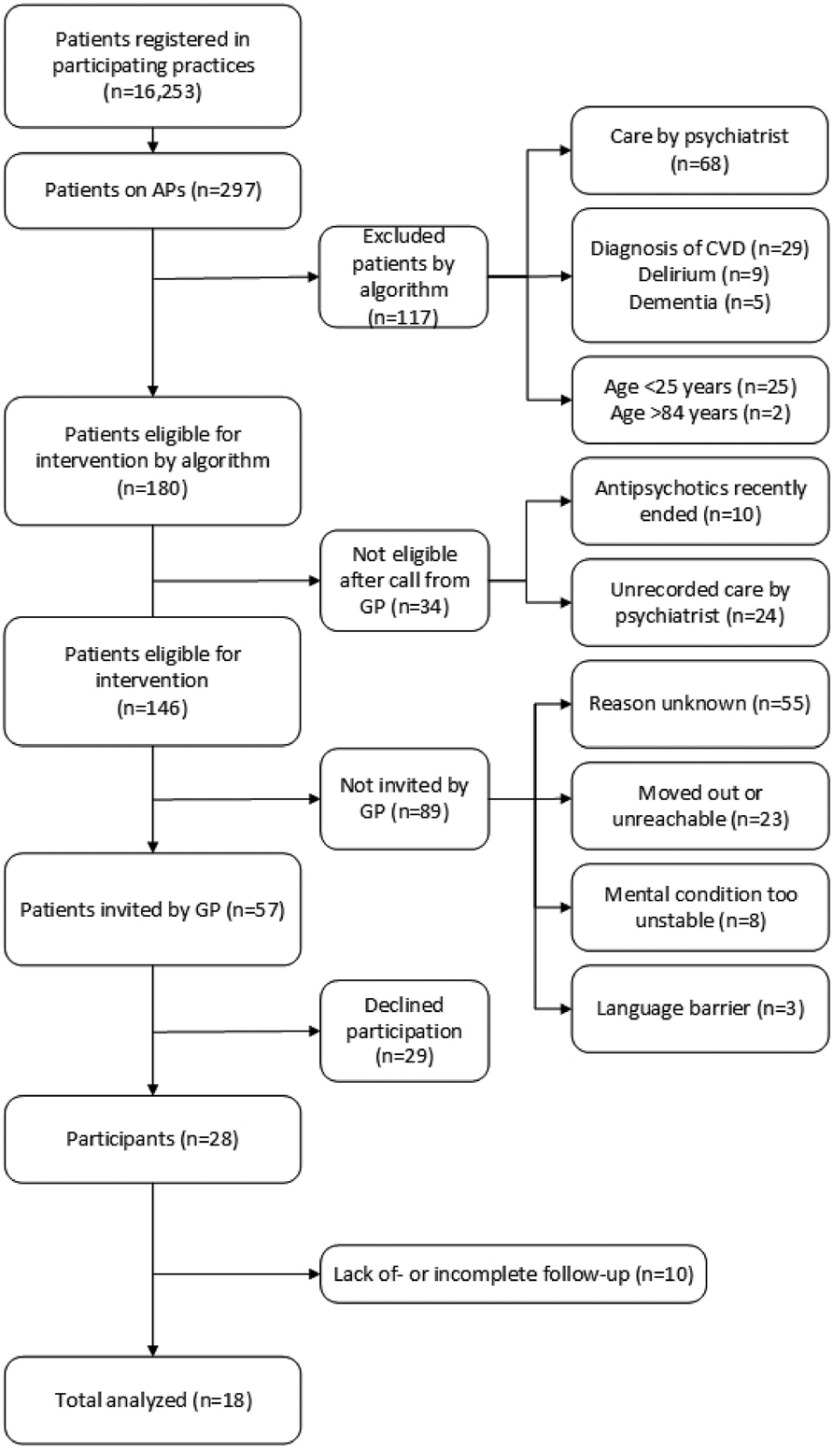
Flow chart of inclusion and exclusion and follow-up of patients. AP, antipsychotic; CVD, cardiovascular disease; GP, general practitioner.

Figure 2 shows the flow chart of included and excluded patients in the pilot practices and the GPs’ reasons for exclusion resulting in 28 participants. No reason was given for approximately 61% (n = 55) of eligible patients. Recruitment was between March 1 and May 1, 2021. It is noticeable that 24 patients were cared for by a psychiatrist without the knowledge of the GP. There was a lack of follow-up or it was incomplete for 36% (n = 10). The details of these cases are shown in Table 3. The dropouts were not associated with changes in AP prescriptions. The data collection ended 4 months after the last multidisciplinary meeting.

**Table 3:** Lack of or incomplete follow-up.

ID	Reason for drop out	Timing in relation to intervention	Advice multidisciplinary meeting	Changes in prescriptions	Diagnoses related to AP prescription
24	Dissatisfaction with step 2	Shortly after step 2	Consider lowering Abilify 15 mg in the future	Ended study therefore no further data	Bipolar disorder
12	Died of cancer	Shortly after step 2	Consider halving dosage Quetiapine	No changes	Anxiety disorder
16	Attempted suicide and admission to clinic	Shortly after step 1			Depressive disorder
27	2 admissions to hospital for dysregulation diabetes mellitus	Between step 1 and 2	Consider lowering Pregabalin	No changes	PTSD*
01	Divorced and became homeless	Between step 3 and follow-up	Due to high anxiety level, lowering Quetiapine is not appropriate. Trial treatment Topiramate 25 mg is an option. Smoking cessation	No changes Quetiapine, started Varenicline	PTSD*
11	Grandmother entered palliative stage	Between step 3 and follow-up	Smoking cessation. Lower dosage Quetiapine from 50 mg to 37.5 mg	Quetiapine was lowered from 50 mg to 25 mg, started Varenicline	Anxiety disorder,Depressive disorder, anorexia
13	4 children who had been placed under guardianship unexpectedly came back home	Between step 1 and 2	Schedule a meeting with all health workers involved	No changes	Borderline personality disorder, ADHD**, sleeping problem
22	Spouse got cancer, palliative trajectory	Between step 3 and follow-up	Risperidone from 1.0 mg to 0.5 mg or switch to Quetiapine 25 mg. If overstrung, then back to Risperidone 1mg	Risperidone was lowered from 1 mg to 0,5 mg	Autism spectrum disorder, attention deficit hyperactivity disorder
26	Left for Morocco	Between step 3 and follow-up	Quetiapine nightmares. Alternatives: Topiramate 25 mg or Mirtazapine	Tried Topiramate, not satisfactorily	Depressive disorder, sleeping problem
28	Missing	Between step 3 and follow-up	Citalopram is relatively high dosed, reduced to 30 mg in a stable period. If that goes well then reduce Olanzapine to 2.5 mg or switch to Haldol or Risperidone	No changes	Bipolar disorder

*ADHD* attention deficit hyperactivity disorder, *AP* antipsychotic, *PTSD* post-traumatic stress disorder.

### Baseline characteristics

**Figure 3:**
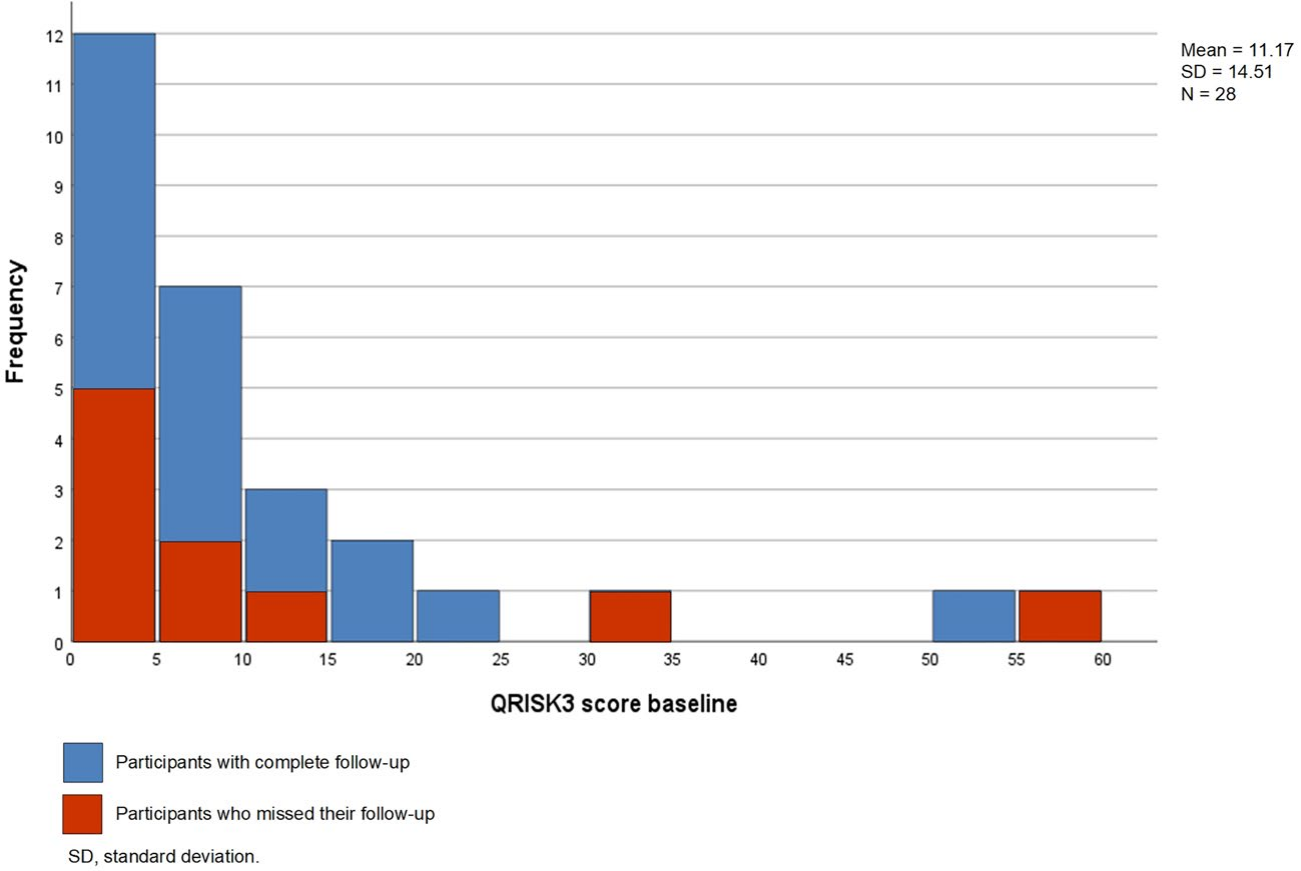
Distribution of QRISK3 score at baseline.

The baseline characteristics of the participants are shown in Table 2. The mean participant age was 49 years (SD = 11). The socioeconomic status was low in 35.7% of participants. The educational level was high in 37%. Quetiapine was the most commonly prescribed AP agent. Only 14% of the participants had a diagnosis of psychosis or bipolar disorder. All participants reported adverse effects. The mean score of the SMS was 22.5 (SD = 10.6), which is categorized as ‘high’. Nine (32%) participants scored ≥ 30, which is categorized as ‘very high’ (should be reported to the prescriber). The mean MHI-5 score was 56.8 (SD = 18.01). The distribution of QRISK3 was positively skewed, as shown in Fig. 3. We did not find a statistical significant or clinical significant difference of mean QRISK3 score between the dropouts and those who had a complete follow-up (Fig. 3).

### Numbers and types of advice given

The intervention was completed by 23 of 28 participants (82%). The multidisciplinary meeting (step 2) generated multiple pieces of advice per patient, based on current insights and guidelines and taking into account the patients’ wishes. Supplement Table S2 shows the type and topic of the advice and whether it was adopted. The majority of the patients were advised to change their AP use immediately or in the future (59% and 19%, respectively). After 3 months, 41% of all advice (28/68) was followed. Out of 10 smokers, eight completed the intervention. Five of eight agreed on smoking cessation during the multidisciplinary meeting, and four of them had quit smoking at the follow-up visit.

### Potential effectiveness of TACTIC

For participants with a complete follow-up, the QRISK3 scores at follow-up were significantly lower than at baseline (Z = − 2.112, p = 0.035). The table in the supplements (Table S3) displays the change in all secondary outcome variables of patients who completed follow-up. The proportional risk reduction is presented in supplements Fig. S2. The mean improvement was 25.4% (n = 18, SD = 58.7). The improvement on the MHI-5 score was not significant (Z = 0.264, p = 0.79). All changes in patient outcomes can be seen in the supplement Table S3. The patients’ satisfaction with the intervention was slightly above neutral (n = 21, mean CSQ-8 score = 23, SD = 5.6).

## Discussion

We assessed the feasibility and the potential health effects of a transmural collaborative care model for patients using APs treated in general practice. This pilot study shows that the intervention is executable in primary care, although it will not reach all eligible patients since many would not participate.

It appeared that 78% of participants were advised to change their use of AP now or in the future. Other advice concerned other medication, lifestyle, monitoring, and psychotherapy. At 3 months, 41% of all advice had been adopted. Of 10 smokers, four had quit smoking (40%). We found a small but significant improvement of the absolute QRISK3 score between baseline and follow-up. This result must be interpreted with caution because of the small number of participants and the high drop-out rate (36%). Dropping-out was never associated with a reduction in AP medications. On the one hand, the participants who were motivated enough to do the follow-up visit were more likely to lower their QRISK3 score. On the other hand, 43% (n = 12) of the participants had no room for improvement on their QRISK3 score because it was already low at baseline and the follow-up time of 3 months was short. Therefore, the significant change seems a promising result.

The main strength of this study is the real-life execution of an innovative and complex intervention that combines the skills of different professionals and has the potential to improve patients’ cardiovascular health in primary care. We learned a lot about characteristics of our target group and pitfalls that should be avoided in the trial.

A principal limitation was the low number of participants. A lot of eligible patients were not invited without a known reason, which could have led to selection bias. This was an unexpected outcome caused by the high workload of the GPs, who were already challenged by the COVID pandemic. We will adjust the inviting routine in such a way that the burden on practices is reduced. Many eligible patients were difficult to reach or unwilling to participate. Former research shows that patients with SMI are a vulnerable group who experience social problems on many often intertwined levels^38,39^. This could make them more difficult to reach, involve, and maintain follow-up. A qualitative study on patient factors that influence access to primary care found that such people often experience unstable housing and do not have a fixed address or telephone number^40^.

A scoping review into cancer screening also found that people with SMI participate less often^41^. Factors involved are psychiatric symptoms, fear, distrust in the health care system, and low priority. Facilitators to participate are support, good health care experiences, and making participation easy^42^.

Of all people who agreed to participate, 36% dropped out before the follow-up visit after 3 months. The reasons for dropout were in accordance with the aforementioned vulnerability to social problems^38,39^. The role of the patient coach with lived experience, to introduce sources of support within the community, can be important during and after the TACTIC intervention.

Our aim was to include patients who are not being treated by a psychiatrist. During the inclusion of patients, we learned that a selection of who is being treated by a psychiatrist based on the correspondence in the EMR is unreliable, because letters from the psychiatrist are often missing. This is in line with an article by van Hasselt et al. in 2015, describing poor communication between Dutch psychiatrists and GPs^43^. Guidelines on communication and responsibilities would be helpful. The NICE guidelines, contrary to the Dutch guidelines, make explicit recommendations regarding referral to secondary care, referral back to primary care, and monitoring and treatment of CVR factors^9^.

Risk-estimation tools such as QRISK3 are not really suited to quantify change in CVR after an intervention. After all, every risk-lowering intervention needs time to reduce atherosclerosis. However, in daily practice, GPs use these tools to explain to patients how much a strategy will help them to lower their risk. American researchers developed an algorithm that resulted in a one-page handout showing the modifiable risk factors to patients with SMI and their clinicians^44^. Patients in practices who used this tool had a 4% relative risk reduction in total modifiable CVR after 12 months compared with patients in control practices^44^. We also compared the QRISK change in modifiable risk factors: the proportional risk. In a consensus meeting, we discussed the use of a relative or absolute measure as primary outcome for the upcoming trial. The conclusion of the meeting was that GPs find a change in absolute risk more convincing because relative risk may obscure the magnitude of the effect on CVR.

The construction of the QRISK3 algorithm causes a skewed distribution. Every risk factor contributes to a higher risk, and fewer people have an accumulation of risk factors. Many people, even in this population, have a QRISK3 score so low that they cannot improve it. A threshold QRISK3 score in the inclusion criteria for the trial will improve efficacy. It will also limit the number of eligible patients for each GP. Presuming that the large group of uninvited patients in this pilot study was the result of a lack of time from the GPs, a tightening of the inclusion criteria for the trial will also benefit feasibility.

Where do we set the QRISK3 threshold? The UK NICE guideline classifies a risk of 10% morbidity and mortality as high^12^. A risk threshold of 10% would have excluded 2/3 of our participants, and mainly the younger ones, because age is a strong contributor in the algorithm. Excluding the young would be undesirable because the QRISK3 algorithm may underpredict risk in young people with psychosis^45^. Besides, the review of APs is equally important for young people. We reached consensus on setting the threshold at ≥ 5% as an additional inclusion criterion for the trial. Hopefully, TACTIC will have a spin-off effect that other patients with APs can benefit from through awareness among physicians and improved collaboration.

In conclusion, this pilot study was essential in preparation for a trial to evaluate health improvement. With a few adjustments, the trial seems expedient and feasible. The room for improvement of treatment appears to be high, given the advice to change the use of AP in 78% of the cases, and it seems possible to decrease CVR in patients using APs in primary care with the TACTIC intervention.

### Supplementary Information

Supplementary Information.

## Data Availability

The datasets generated during and/or analyzed during the current study are available from the corresponding author on reasonable request.
