# Author Correction: Transmural collaborative care model for the review of antipsychotics: a feasibility study of a complex intervention

**DOI:** 10.1038/s41598-024-69477-2

**Published:** 2024-08-12

**Authors:** Kirsti M. Jakobs, Karlijn J. van den Brule‑Barnhoorn, Jan van Lieshout, Joost G. E. Janzing, Wiepke Cahn, Maria van den Muijsenbergh, Marion C. J. Biermans, Erik W. M. A. Bischoff

**Affiliations:** 1 Primary and Community Care Department Nijmegen, Radboud University Medical Center, Nijmegen, The Netherlands; 2 Zorggroep Onze Huisartsen, Arnhem, the Netherlands; 3 IQ Health Science Department, Radboud University Medical Center, Nijmegen, The Netherlands; 4 Psychiatry Department, Radboud University Medical Center, Nijmegen, The Netherlands; 5 Psychiatry Department, University Medical Center Utrecht, Utrecht, The Netherlands; 6 Pharos, Dutch Centre of Expertise On Health Disparities, Utrecht, The Netherlands

Correction to: *Scientific Reports* 10.1038/s41598-024-62349-9, published online 29 May 2024

The original version of this Article contained an error in Figure 3, where the number of participants who missed their follow-up was incorrect.

The original Figure 3 and accompanying legend appear below.

**Figure 3:**
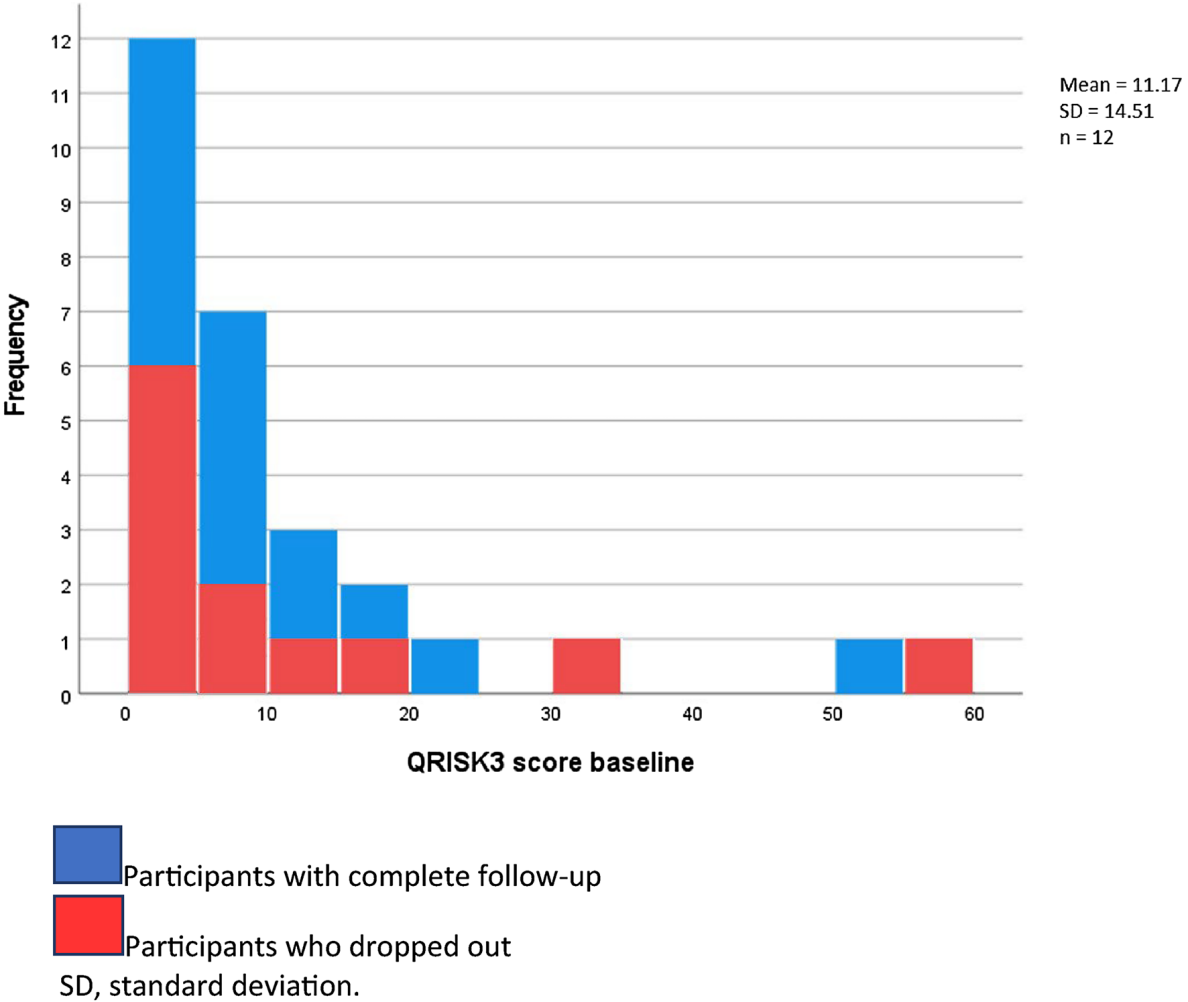
Distribution of QRISK3 score at baseline.

The original Article has been corrected.

