# Supplementary material for: Transmural collaborative care model for the review of antipsychotics: a feasibility study of a complex intervention
Source: Sci Rep. 2024 May 29;14:12367. doi: 10.1038/s41598-024-62349-9 (PMC11137011; doi:10.1038/s41598-024-62349-9)

**Supplement to Transmural collaborative care model for the review of antipsychotics: a feasibility study of a complex intervention**

Authers

Kirsti M. Jakobs*^1,2^, Karlijn J. van den Brule-Barnhoorn^1^, Jan van Lieshout^3^, Joost G.E. Janzing^4^, Wiepke Cahn^5^, Maria van den Muijsenbergh^1,6^, Marion C.J. Biermans^1^ & Erik W.M.A. Bischoff^1^

1 Radboud University Medical Center, Primary and Community Care Department Nijmegen, the Netherlands. 2 Zorggroep Onze Huisartsen, Arnhem, the Netherlands. 3 Radboud University Medical Center, IQ Health Science Department, Nijmegen, the Netherlands. 4 Radboud University Medical Center, Psychiatry Department, Nijmegen, the Netherlands. 5 University Medical Center Utrecht, Psychiatry Department. 6 Pharos, Dutch Centre of Expertise on Health Disparities, Utrecht, the Netherlands.

**Table of contents Page**

[**Supplementary Introduction** 2](#_Toc164963729)

[Project description “PLEK voor EPA” 2](#_Toc164963730)

[Figure.S1 Flow-chart multidisciplinary meeting 3](#_Toc164963731)

[**Supplementary Methods** 4](#_Toc164963732)

[Table S1. Adjustments necessary to calculate a change in QRISK3 score. 4](#_Toc164963733)

[**Supplementary Results** 5](#_Toc164963734)

[Table S2. Overview of advice given during the multidisciplinary meetings. 5](#_Toc164963735)

[Table S3. Changes in patient outcome in patients who completed follow-up (n = 18 unless mentioned otherwise). 5](#_Toc164963736)

[Additional analyses on QRISK3 6](#_Toc164963737)

[Figure S2. Distribution of proportional QRISK3 reduction. 7](#_Toc164963738)

# **Supplementary Introduction**

# Project description “PLEK voor EPA”

There are still significant gaps in the areas of health, relationships, participation, and personal recovery among people with severe mental illness. In the Arnhem region, since 2016, general practitioners and their nurses, psychiatrists, nurses from mental health institutions, experts with lived experience, and representatives of the municipality have been working together to improve collaboration around these patients. The collaboration was tested in two pilot projects called “PLEK voor EPA”. Patients were discussed in a multidisciplinary consultation where they, often with their loved ones or supervisors, were actively involved.

In one pilot project, the use of antipsychotics was a central topic, where the pros and cons were carefully considered. Every patient received personalized advice.

In the other pilot project, the focus was on discussing the wishes and opportunities for recovery care in the neighborhood. The goal was to enhance the quality of life through personal recovery.

Both pilot projects demonstrated how somatic and psychosocial care can be improved together for these individuals. The results were a description of the process, that can serve as an inspiration for others to follow.

Figure.S1 Flow-chart multidisciplinary meeting
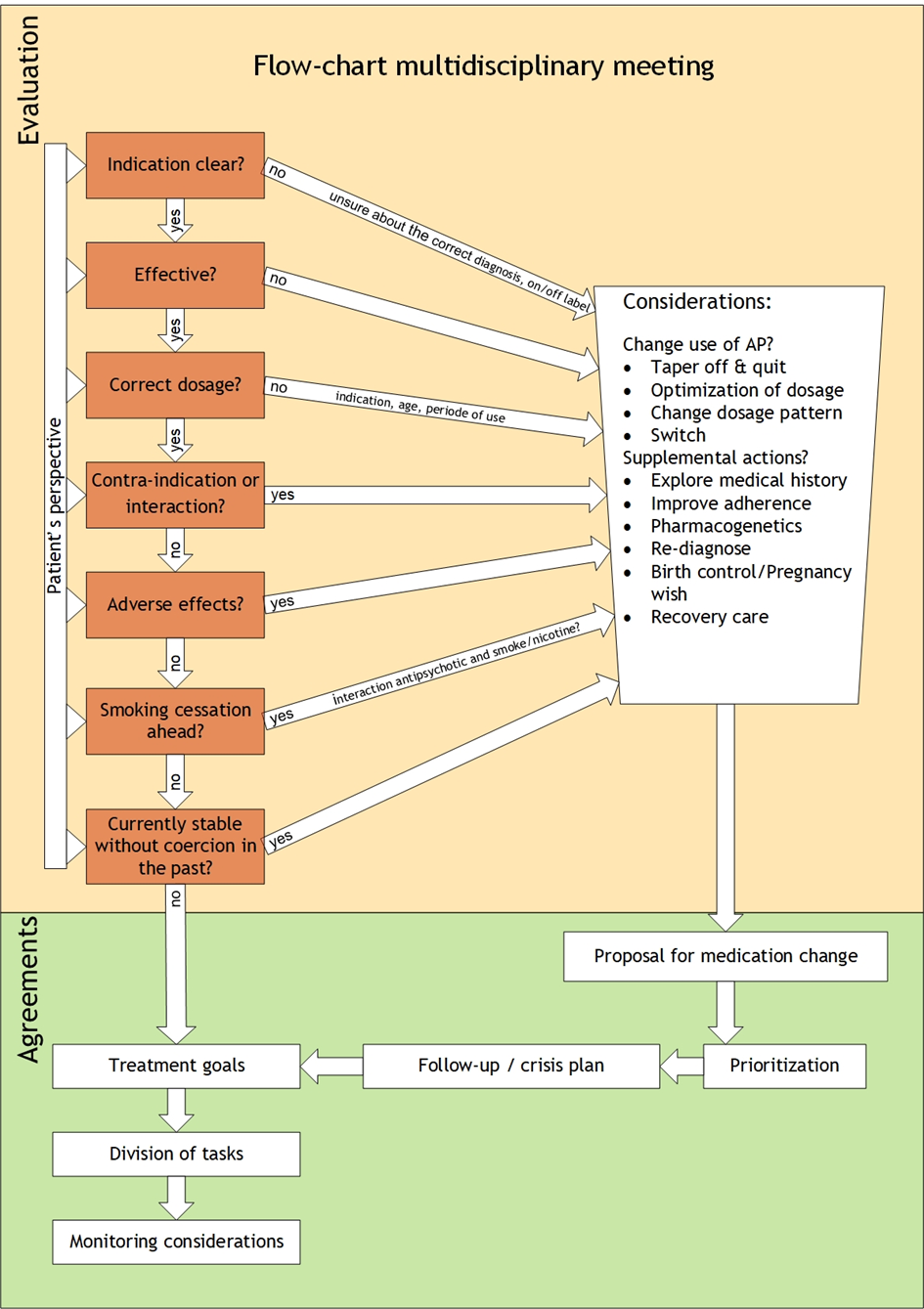


# **Supplementary Methods**

## Table S1. Adjustments necessary to calculate a change in QRISK3 score.

| **Risk factor** | **Value at follow-up** |
| --- | --- |
| Family history of premature coronary heart disease in a first-degree relative* | If yes,  value at baseline is leveled to follow-up value |
| Antihypertensive medication** | If no,  value at baseline is used as follow-up value |
| Age | Leveled to baseline |

*This factor might not be known at the start of enrolment but does contribute to the patient’s cardiovascular risk.

**If ‘on blood pressure treatment’ is answered ‘yes’, this will raise the QRISK3 score according to the algorithm. In case a patient starts blood pressure treatment due to the intervention, this will be ignored at follow-up.

# **Supplementary Results**

## Table S2. Overview of advice given during the multidisciplinary meetings.


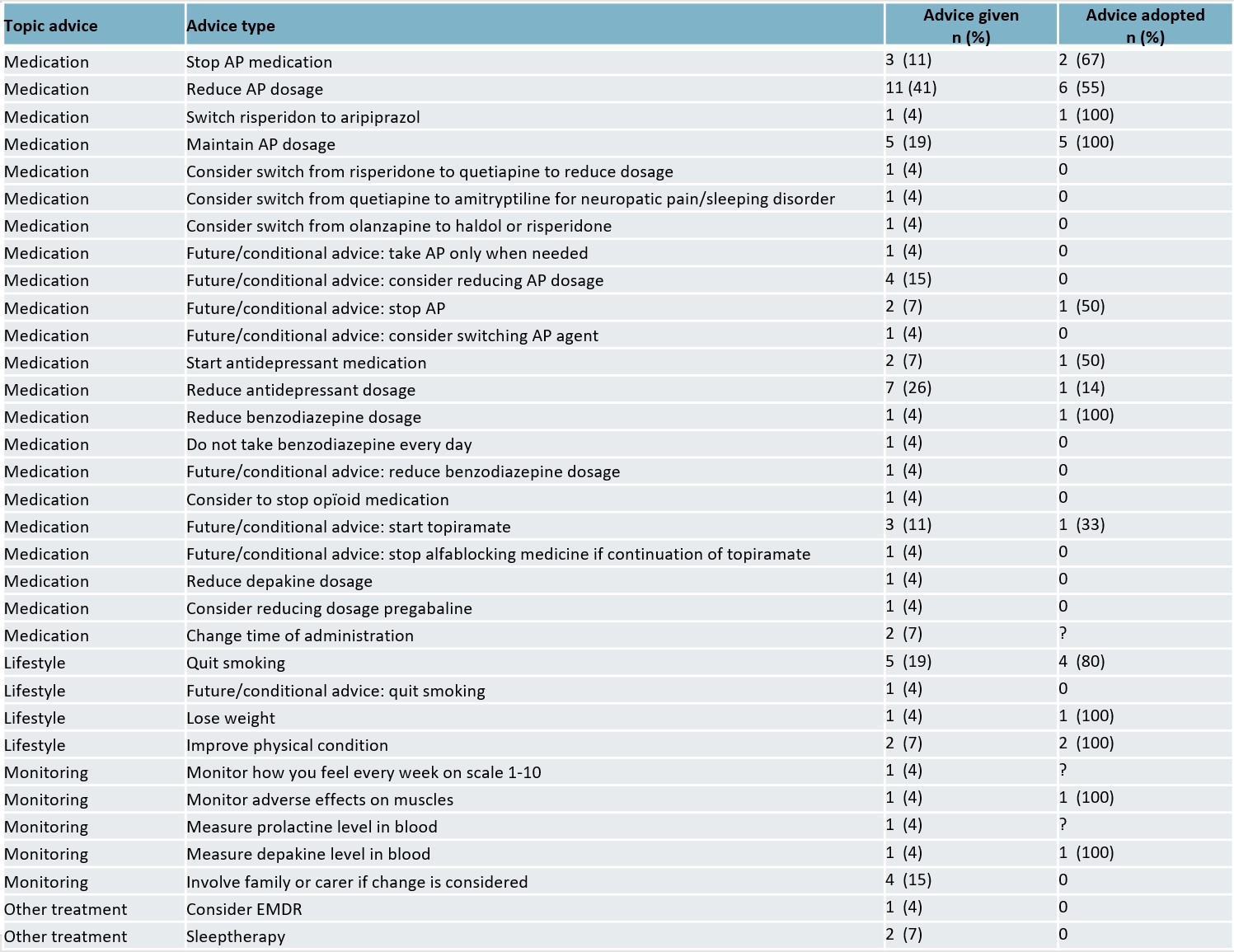


AP, antipsychotic; EMDR, eye movement desensitization and reprocessing.

## Table S3. Changes in all secondary outcome variables of patients who completed follow-up (n = 18 unless mentioned otherwise).

| **QRISK3 variable** | **T0 (SD)** | **T1 (SD)** | **T0–T1 (SD)** |
| --- | --- | --- | --- |
| Mean QRISK3 score | 10.29 (12.55) | 8.96 (11.10) | 1.32 (2.84) |
| Mean QRISK3 score with TDS | 10.99 (12.78) | 9.47 (11.13) | 1.52 (3.42) |
| Mean cholesterol ratio | 4.05 (1.32) | 4.10 (1.39) | –0.05 (0.40) |
| Mean systolic blood pressure | 127.00 (19.43) | 121.33 (14.58) | 5.67 (9.34) |
| Mean SD of at least two most recent systolic blood pressure readings | 6.83 (6.48) | 6.27 (6.35) | –2.22 (4.52) |
| Mean BMI | 26.98 (4.84) | 26.83 (4.36) | 0.14 (1.21) |
| Albumin creatinine ratio | 0.82 (3.28) | 0.31 (0.75) | 0.64 (3.36) |
| Antihypertensive treatment | 1 | 2 | 1 |
| APs | 18 | 15 | 3 |
| Chronic corticosteroids | 1 | 1 | 0 |
| Chronic kidney disease stage 3–5 | 2 | 0 | 2 |
| **Mental health variable** **(n=21)** | **T0** | **T1** | **T0–T1** |
| MHI-5 scores (SD) | 59.52 (17.31) | 61.43 (18.98) | –1.90 (15.04) |
| **Smoking* (n=22)** | **T0** | **T1** | **T0–T1** |
| Never | 7 | 7 | 0 |
| Past | 8 | 12 | –4 |
| Light | 4 | 3 | 1 |
| Medium | 3 | 0 | 3 |
| Heavy | 0 | 0 | 0 |
| **Adverse effects (n=21)** | **T0** | **T1** | **T0–T1** |
| Mean adverse effects (SD) | 22.57 (11.65) | 17.33 (10.30) | 5.24 (5.04) |
| **EQ-5D (n=21)** | **T0** | **T1** | **T0–T1** |
| Mean EQ-5D | 0.33 (0.29) | 0.42 (0.28) | –0.09 (0.24) |

* Data derived from EMR and questionnaires.

AP, antipsychotic; BMI, body mass index; EQ-5D, generic quality of life; MHI, Mental Health Inventory; SD, standard deviation; TDS, Townsend deprivation score.

## Additional analyses on QRISK3

The distribution of the proportional reduction in QRISK3 score, being the difference in QRISK3 score resulting from the intervention as a proportion of what could be achieved (a QRISK3 score with all modifiable risk factors optimized), is shown in Figure S2. For the 18 participants who had a complete follow-up, the risk reduction is presented on the x-axis. The mean improvement was 25.4% (SD=58.7). The proportional changes of three participants are outliers exceeding (–)100%. These participants had a very low maximum achievable improvement. A slight change in cholesterol ratio the wrong way (within the optimum range) or an improvement of systolic blood pressure (lower than what was defined as optimal) caused these outliers.

## Figure S2. Distribution of proportional QRISK3 reduction.


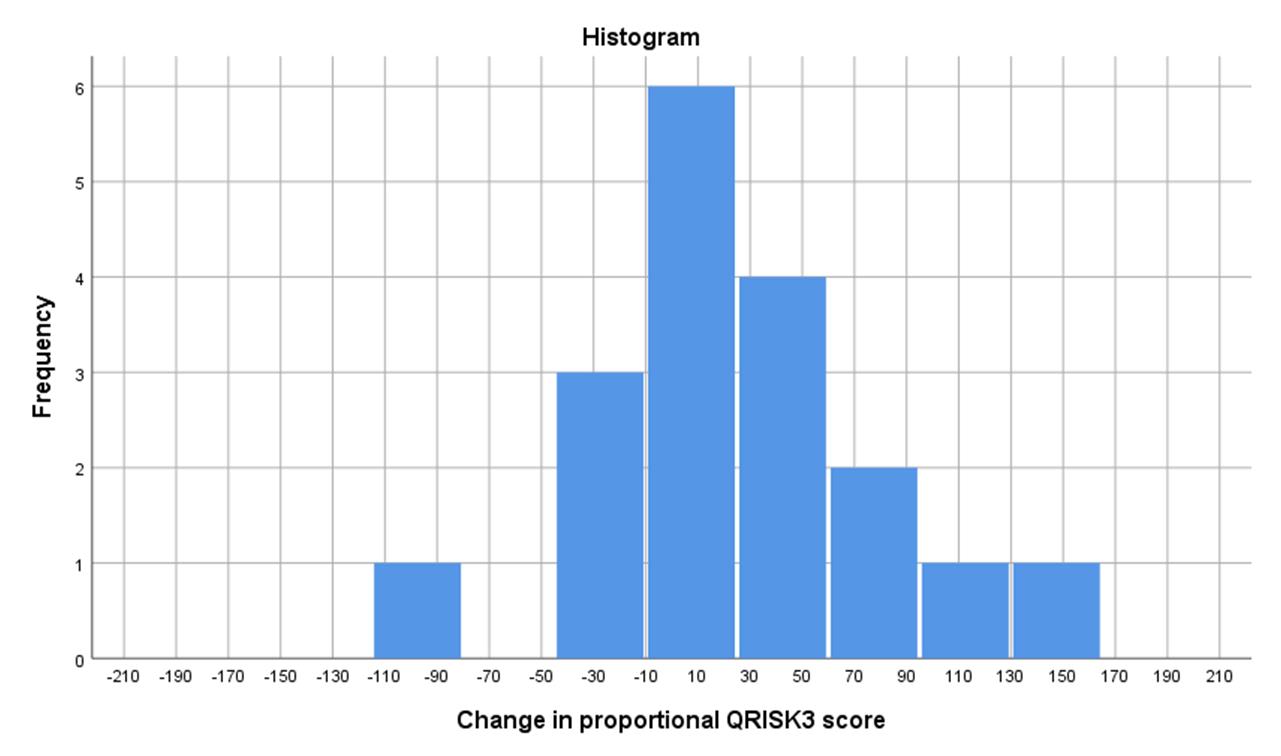

Supplement: Supplementary file 1 — Supplementary Information. [file 41598_2024_62349_MOESM1_ESM.docx]
